# Effects of Estrogen Fluctuation during the Menstrual Cycle on the Response to Stretch-Shortening Exercise in Females

**DOI:** 10.1155/2013/243572

**Published:** 2013-09-12

**Authors:** Saulė Sipavičienė, Laura Daniusevičiutė, Irina Klizienė, Sigitas Kamandulis, Albertas Skurvydas

**Affiliations:** ^1^Department of Applied Physiology and Kinesiotherapy, Lithuanian Sports University, Sporto 6, 44221 Kaunas, Lithuania; ^2^Department of Physical Education, Kaunas University of Technology, K. Donelaičio g. 73, LT-44029 Kaunas, Lithuania; ^3^Research Center for Fundamental and Clinical Movement Science, Lithuanian Sports University, Sporto 6, 44221 Kaunas, Lithuania

## Abstract

The aim of this study was to investigate whether variation in estrogen levels during the menstrual cycle influences susceptibility to exercise-induced muscle damage after stretch-shortening cycle exercise. Physically active women (*n* = 18; age = 20.2 ± 1.7 yr) participated in this research. The subjects performed one session of 100 maximal drop jumps on day 1 or 2 of the follicular phase and another identical session on day 1 or 2 of the ovulatory phase; the order of the sessions was randomized. Quadriceps femoris muscle peak torque evoked by electrical stimulation and maximal voluntary contraction, muscle pain, and CK activity were measured before and at various times up to 72 h after exercise. It was found that the high estrogen level during the ovulatory phase might be related to an earlier return to baseline muscle strength after strenuous stretch-shortening cycle exercise in that phase compared with the follicular phase. The estrogen effect appears to be highly specific to the damaged site because the differences in most EIMD markers (CK, soreness, and low-frequency fatigue) between the two menstrual cycle phases were small.

## 1. Introduction

An unaccustomed and intensive or high-volume physical load frequently produces exercise-induced muscle damage (EIMD) and delayed muscle soreness [1, 2]. This is particularly common when performing stretch-shortening cycle exercises involving lengthening of an active muscle followed by rapid concentric contraction [3]. The soreness and swelling of muscles, increased plasma creatine kinase (CK) activity, and decline in and slow recovery of muscle strength are considered indirect markers of EIMD [4]. EIMD has been reported in both human and animal studies [1, 4–7].

In humans, the extent of muscle damage differs between individuals and can be explained by different factors such as the type and intensity of exercise and the person's age, fitness level, and genotype [8–10]. Sex differences and, more precisely, hormonal differences may influence the extent of damage and recovery in muscles. Research on rats has suggested that the female hormone estrogen influences the post-EIMD repair of skeletal muscle through activation of satellite cells [11]. In support of this concept, female animals exhibit less muscle damage than do male animals [7, 12]. Thus, it is believed that sex hormones may influence the extent of muscle damage and recovery after EIMD [13].

In contrast to studies of animals, studies of humans have not clearly defined the effect of estrogen on muscle contractile function and EIMD [14]. There is no uniform opinion, and some researchers maintain that estrogens do not exert a significant effect on muscle damage after a physical load [15], whereas others claim that sex hormones can mitigate muscle damage and enhance recovery [13, 16]. Although sex differences in EIMD have been researched in animal models, insufficient research has been performed in humans. Most research on sex differences in muscle fatigue and recovery has not taken into account the phases of the menstrual cycle, during which estrogen levels fluctuate considerably [7, 17, 18]. Although data from human studies are contradictory [19, 20], results from animal studies lead us to hypothesize that less muscle damage would occur after a physical load and that the muscle recovery would be faster during the menstrual cycle phase with the highest concentration of estrogens.

The aim of this study was to investigate whether variation in estrogen level during the menstrual cycleinfluences a woman's susceptibility to EIMD in response to stretch-shortening cycle exercise. Because estrogen levels differ significantly between the early follicular and ovulatory phases of the menstrual cycle [17], we compared the responses to stretch-shortening exercise performed during these two phases.

## 2. Material and Methods

### 2.1. Participants

Healthy, physically active women (*n* = 18; age = 20.2 ± 1.7 yr; weight = 56.2 ± 4.1 kg; height = 167.3 ± 3.2 cm) not using hormone contraception and with a regular menstrual cycle participated in this research. To assess the menstrual cycle phases, rectal temperature was recorded every morning before arising from bed for three consecutive months. The beginning of the follicular phase was indicated by the onset of menses, and the beginning of ovulation was indicated by an increase in temperature of 0.5°C. The research protocol was discussed with and approved by the Kaunas Regional Biomedical Research Ethics Committee. Before the investigation began, each subject read and signed a written informed consent form, and the study protocol was consistent with the principles outlined in the Declaration of Helsinki.

### 2.2. Performance of Stretch-Shortening Cycle Exercise Load

Each subject performed 100 maximum drop jumps from a height of 0.75 m from a platform with an immediate maximum rebound on a Kistler force plate (type 9286A, Amherst, NY, USA). Jumps were performed with a countermovement to a 90° angle in the knee at an interval of 20 s. While performing the jumps, the subject placed the hands on the waist. The subjects were informed of the height of each jump and were motivated to perform each jump as high as possible. The height of all jumps was recorded, and the difference between the highest of the 10 initial jumps and the highest of the 10 final jumps was used as an indicator of the fatigue induced by the stretch-shortening cycle exercise.

### 2.3. Isometric Torque and Electrical Stimulation

Isometric torque was measured in the knee extensor muscles using an isokinetic dynamometer (System 3; Biodex Medical Systems, Shirley, NY, USA). Direct muscle stimulation was applied using three carbonized rubber electrodes covered with a thin layer of electrode gel (ECG-EEG Gel; Medigel, Modi'in, Israel). Two of the electrodes (6 × 10 cm) were placed transversely across the width of the proximal portion of the quadriceps femoris. The third electrode (6 × 10 cm) covered the distal portion of the muscle above the patella. A standard electrical stimulator (MG 440; Medicor, Budapest, Hungary) was used. The electrical stimulation was delivered in square-wave pulses of 1 ms duration. The tolerance of the volunteers to electrical stimulation was assessed on a separate occasion, and all participants who were recruited for the study showed good compliance with the procedure. The intensity of electrical stimulation was selected individually by applying single stimulus to the muscles tested. During electrical stimulation, the leg was fixed at a knee angle of 90° (0° = full knee extension). The measurements included maximal voluntary isometric contraction (MVC) torque and the torque evoked in the quadriceps by 1 s trains of electrical stimulation at 20 Hz (P20) and 100 Hz (P100). The procedures for electrical stimulation were essentially the same as those described previously [21].

### 2.4. Plasma CK Activity

About 5 mL of blood was drawn from the median cubital vein by experienced medical personnel. Samples were centrifuged immediately and analyzed for CK activity using a Spotchem EZ SP-4430 biochemical analyzer (Menarini Diagnostics, Winnersh-Wokingham, UK) with soft reagent strips (ARKRAY Factory, Inc., Shiga, Japan).

### 2.5. Concentration of Estrogen in Blood

At the beginning of each experiment, a 5 mL sample of venous blood was taken to measure the estrogen (17*β*-estradiol) concentration in blood. The blood was analyzed using electrochemiluminescence analysis and the Roche Elecsys 1010/2010 Cobas e 411 and Modular Analytics E170 immunological analyzers (Roche Diagnostics GmbH, Germany).

### 2.6. Muscle Soreness

The severity of the soreness of the quadriceps was rated subjectively by subjects after 2-3 squats using a 0–10-point scale. This muscle soreness evaluation method has been used in previous studies [6, 22].

### 2.7. Experimental Procedure

One week before the research, the subjects were acquainted with the course of the experiment and were taught how to perform the exercise. The subjects performed the experimental protocol twice: (1) one session on day 1 or 2 of the follicular phase and (2) another session on day 1 or 2 of the ovulatory phase. The order of these sessions was randomized and time interval between sessions was at least 10 weeks to minimize potential manifestation of repeated bout effect [23]. On the day of the experiment in both phases, a sample of venous blood was taken for measurement of 17*β*-estradiol concentration and CK activity. The subject performed a warmup of running in place at low intensity for 5 min, after which she then sat on the chair of the Biodex dynamometer. During testing, the subject performed a MVC and the peak torque of an involuntary contraction of the quadriceps femoris muscle was evoked by electrical stimulation at 20 Hz and 100 Hz frequency. The subject then performed a stretch-shortening cycle exercise workout to evoke quadriceps femoris muscle damage (as described above). Muscle strength was measured again at 2 min and 24, 48, and 72 h after the workout. Muscle pain was assessed at 24, 48, and 72 h, and CK activity at 24 h after the workout.

### 2.8. Statistical Analysis

We calculated the arithmetic average and the standard deviation for each variable. Normality of data distribution was tested and confirmed by the Kolmogorov-Smirnov test. A two-way analysis of variance (ANOVA) for repeated measures was used to determine the effects of menstrual phase (follicular versus ovulatory phase) and time (after 2 min and 24, 48, and 72 h of recovery) on the measured properties in quadriceps muscle. If a significant effect was found, a post hoc test was performed to locate the differences between means by applying paired *t* tests with a Bonferroni correction for multiple comparisons. Changes in jump height, CK activity, and muscle soreness were analyzed by paired *t*-tests. Significance was set at *P* < 0.05.

## 3. Results

### 3.1. Initial Values

17*β*-estradiol concentration was significantly higher in the ovulatory than in the follicular phase (229.6 ± 81.8 pmol/L versus 119 ± 61.7 pmol/L, *P* < 0.05). However, MVC and electrically induced muscle contraction torque did not differ between phases (*P* < 0.05, Table 1).

### 3.2. Stretch-Shortening Cycle Exercise

The jump height decreased throughout the exercise bout in both menstrual cycle phases. When comparing the best attempt of the 10 initial with the 10 final jumps, the height decreased by 14.5 ± 3.5% in the follicular phase and by 9.1 ± 2.1% in the ovulatory phase (*P* > 0.05).

### 3.3. Muscle Strength

**Figure 1:**
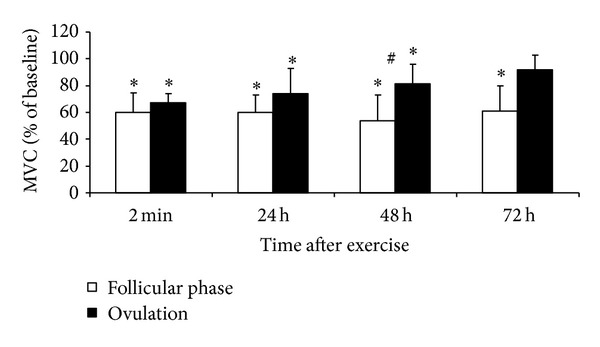
Time course of changes in the maximal voluntary contraction (MVC) torque (mean ± SD) after stretch-shortening cycle exercise. *P < 0.05—compared to initial level. ^#^ *P* < 0.05—between follicular phase and ovulation.

**Figure 2:**
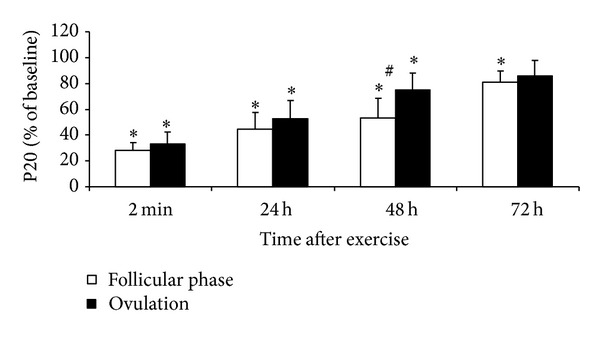
Time course of changes in torque (mean ± SD) evoked in quadriceps by 1 s trains of electrical stimulation at 20 Hz (P20) after stretch-shortening cycle exercise. *P < 0.05—compared to initial level. ^#^ *P* < 0.05—between follicular phase and ovulation.

**Figure 3:**
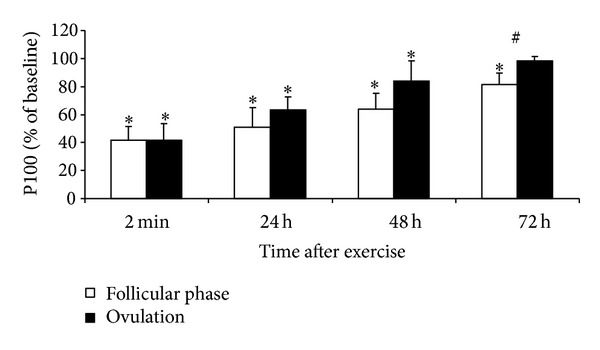
Time course of changes in torque (mean ± SD) evoked in quadriceps by 1 s trains of electrical stimulation at 100 Hz (P100) after stretch-shortening cycle exercise. *P < 0.05—compared to initial level. ^#^ *P* < 0.05—between follicular phase and ovulation.

MVC and electrically induced knee extension torque decreased significantly immediately after the stretch-shortening cycle exercise in both menstrual phases (*P* < 0.05 compared with the respective initial level, *P* > 0.05 between phases, Figures 1, 2, and 3). 

**Figure 4:**
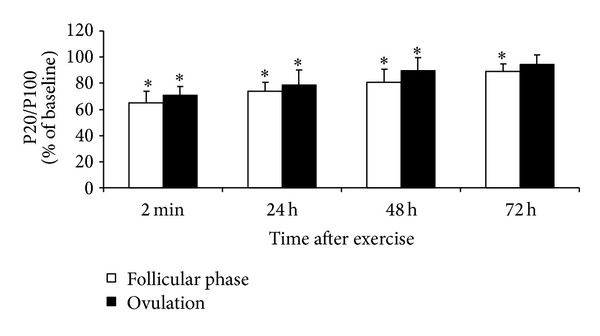
Time course of changes in P20/P100 (mean ± SD) after stretch-shortening cycle exercise. *P < 0.05—compared to initial level.

MVC and P100 measured 48 and 72 h after exercise were lower in the ovulatory than in the follicular phase (*P* < 0.05). P20 did not differ between phases. MVC, P20, and P100 recovered completely at 72 h after exercise in the ovulatory phase but remained reduced at this time in the follicular phase (*P* < 0.05 compared with initial level). The P20/P100 ratio decreased in both conditions (*P* < 0.05) but did not differ between conditions at any time point (Figure 4).

### 3.4. CK Activity and Muscle Soreness

**Figure 5:**
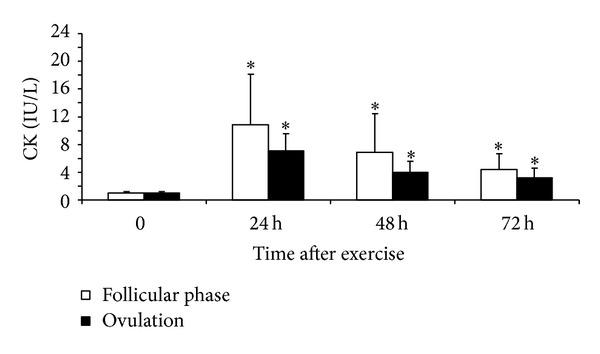
CK activity (mean ± SD) before (0) and 24, 48, and 72 hours after stretch-shortening cycle exercise. *P < 0.05—compared to initial level.

**Figure 6:**
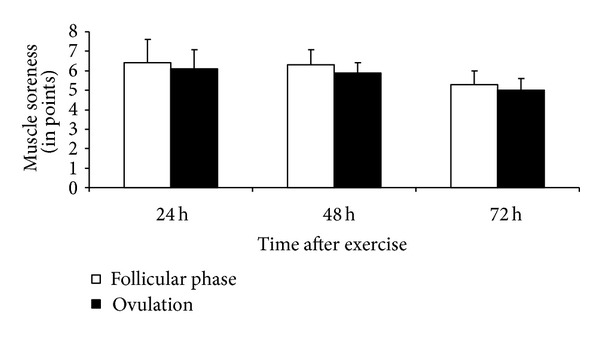
Muscle soreness (mean ± SD) after 24, 48, and 72 hours of stretch-shortening cycle exercise.

After stretch-shortening cycle exercise, CK activity increased to a similar level in both phases (*P* < 0.05 compared with the respective initial level, *P* > 0.05 between phases, Figure 5). Muscle soreness did not differ between the two phases at any time point after exercise (*P* > 0.05, Figure 6).

## 4. Discussion 

The results of this research demonstrate two points. First, indirect indicators of muscle damage, such as the decreases in MVC, electrically induced knee extension torques, and low-frequency fatigue measured immediately after stretch-shortening cycle exercise, did not differ between phases of the menstrual phase. Second, quadriceps muscle strength recovery was greater in the ovulatory compared with the follicular phase, whereas low-frequency fatigue, CK activity, and muscle soreness did not differ between phases.

*EIMD.* The experimental conditions used in this study appeared to cause moderate-to-severe EIMD, as shown by the large reduction in force-generating capacity, especially with low-frequency stimulation (>70%), muscle pain (6-7 points out of 10), and 5-6-fold increase in plasma CK activity. The decrease in muscle strength immediately after stretch-shortening cycle exercise does not appear to be related to the accumulation of metabolites, such as phosphate and hydrogen ions, in the myoplasm because drop jumps performed at 20 s intervals are unlikely to induce significant changes in energy metabolites [24]. In addition, the reduction in muscle strength remained evident for at least 2 days of recovery, which is much too long for metabolites to affect muscle function. The observed changes in muscle performance following the stretch-shortening exercise may reflect structural changes in the contractile and cytoskeletal elements within muscle fibers. Such structural abnormalities in the muscle after eccentric exercise may include irregular Z-disks, disorganized myofilaments, and hypercontracted or overstretched sarcomeres [1, 25]. Structural changes may also reflect the remodeling of the myofibrillar structure [26, 27].

Larger reductions in evoked torque were observed at 20 Hz compared with 100 Hz muscle stimulation, indicating the presence of low-frequency fatigue after 100 jumps (Figure 4). The decrease in muscle torque evoked by low-frequency stimulation reflects a decrease in the concentration of Ca^2+^ ions discharged from the sarcoplasmic reticulum and the sensitivity of myofibrils to Ca^2+^ ions [28]. Because of differences in excitation-contraction coupling dynamics, the muscle torque evoked by high-frequency stimulation is likely to change less than that evoked by low-frequency stimulation [29, 30]. The observed low-frequency fatigue in the present study may reflect a failure in excitation-contraction coupling.

*Menstrual Cycle Effect.* The data partly confirm our research hypotheses that, at high estrogen concentrations, EIMD immediately after strenuous exercise might be less severe and muscle recovery might be more rapid. In these subjects, the similar degree of impairment of muscle function (e.g., decreased torque and jump height) immediately after jumping suggests a similar amount of damage induced by strenuous stretch-shortening cycle exercise (Figures 1–3). These findings contrast with the results observed during the recovery period when muscle strength recuperated to the initial level earlier in the ovulatory than in the follicular phase. This difference strongly suggests that specific menstrual cycle functional changes are related to the effect of estrogen on the muscle regeneration mechanisms and that regeneration after EIMD may be associated with the inflammatory response to muscle-damaging exercise. Some research in animals has shown that estrogens directly affect inflammation and that estrogen decreases leukocyte infiltration into muscles after novel exercise [7, 9]. Prevention of leukocyte infiltration by estrogens may restrict the damage and elicit faster muscle recovery. Muscles may recover from EIMD faster in females because of their higher estrogen levels and a weaker inflammatory reaction [15, 31]. However, this particular effect of estrogens is yet to be documented in humans [31].

The infiltration into muscles of leukocytes (especially macrophages), which activate satellite cells, is essential to muscle regeneration [32, 33]. Thus, prevention of leukocyte infiltration into muscles may slow important stages in the muscle regeneration process [7]. Inflammation may also decrease the damage to muscle by reducing the production of oxidizing factors. For example, estrogens may affect the activity of inducible nitric oxide synthase (iNOS) and the inductive synthesis of iNOS in multiple tissues and may thus affect the extent of damage to and recovery of muscles after exercise [34]. Estrogens released in response to skeletal muscle damage may also affect the inflammatory and muscle-regeneration processes by influencing the production of iNOS in satellite cells within muscles [9, 11].

Even though the release of CK from muscles is not a direct indicator of the damage to muscle structure, it is considered an adequate indicator of impaired stability of the muscle cell membrane [4]. Increased permeability of the sarcolemma associated with muscle damage allows CK to exit the muscles and appear in the bloodstream [35]. Estrogens are strong antioxidants that function as membrane stabilizers by interacting with the phospholipid double layer of the membrane [9]. These antioxidant qualities of estrogens might mitigate the muscle damage after a physical load [13]. We did not explore whether the extent of damage to the muscle cell membrane associated with EIMD changes during the menstrual cycle. Although CK activity provides an estimation of the magnitude of the damage, changes in CK activity should be interpreted with caution because the CK level can be influenced by the rate of removal from the bloodstream [36] or previous exposure to eccentric-type exercise [22].

After excessive eccentric physical loads, the damaged cells release bradykinin, histamine, and prostaglandins, which increase the sensitivity of the pain receptors and contribute to delayed muscular soreness and sensitivity [37]. Soreness commonly appears together with other typical symptoms of the inflammatory process such as muscle stiffness, swelling, and sensitivity to palpation [4]. Muscle soreness was also observed in the present study but did not differ significantly between menstrual cycle phases. Consistent with the changes in muscle soreness, the changes in low-frequency fatigue were independent of estrogen level in the present study. This suggests that the estrogen-mediated effect was insufficient to produce changes in muscle soreness and the low-frequency fatigue-generating mechanisms.

The present study indicates that increase in estrogen level does affect the muscle regeneration process and does improve muscle function during the ovulatory phase. However, we note that other important factors besides estrogens (e.g., fluid distribution and thermoregulation) may influence the recovery after physical load during different menstrual phases [17]. The lack of a “gold standard” experimental model is a limitation in all human studies of estrogen effects on muscle function. 

## 5. Conclusions

The present study demonstrates that muscle strength returns to the baseline level faster after strenuous stretch-shortening cycle exercise during the ovulatory phase, when the estrogen level is high, compared with the follicular phase. The estrogen effect appears to be highly specific to the damaged site because the differences in most EIMD markers (CK, soreness and low-frequency fatigue) between the two menstrual cycle phases were small.

## Figures and Tables

**Table 1:** The initial values (mean ± SD) of voluntary and involuntary performance in follicular phase and ovulation.

Menstrual cycle	Jump height, cm	MVC, Nm	P20, Nm	P100, Nm
Follicular phase	21.2 ± 2.7	130.3 ± 22.1	53.1 ± 11.9	76.3.±29.9
Ovulation	22.1 ± 2.5	137.1 ± 28.6	57.9 ± 21.6	78.3 ± 21.1

MVC: maximal voluntary contraction torque; P20: torque evoked in quadriceps by 1 s trains of electrical stimulation at 20 Hz; P100: torque evoked in quadriceps by 1 s trains of electrical stimulation at 100 Hz.
